# Prevalence and Main Determinants of BRAF V600E Mutation in Dysplastic and Congenital Nevi

**DOI:** 10.30699/ijp.2020.130968.2451

**Published:** 2020-10-10

**Authors:** Alierza Ghanadan, Tahereh Yousefi, Kambiz Kamyab-Hesari, Vahidehsadat Azhari, Maryam Nasimi

**Affiliations:** 1 *Department of dermatopathology, Razi hospital, and pathology department of cancer institute, Imam Khomeini hospital complex, Tehran University of Medical Sciences, Tehran, Iran*; 2 *Department of Anatomical Pathology, Yas hospital complex, Tehran University of Medical Sciences, Tehran, Iran*; 3 *Department of dermatopathology, Razi hospital, Tehran University of Medical Sciences, Tehran, Iran*; 4 *Department of Dermatology, Razi Hospital, Tehran University of Medical Sciences, Tehran, Iran*

**Keywords:** BRAF mutation, Congenital nevi, Dysplastic nevi

## Abstract

**Background & Objective:**

Predicting the transformation of dysplastic or congenital nevi into malignant lesions results in a significant increase in the survival of patients. Some specific gene mutations have been reported to be very helpful in this regard. Therefore, this study aimed to evaluate the prevalence of BRAF V600E mutation in dysplastic and congenital nevi.

**Methods:**

This cross-sectional study was conducted on patients with congenital (n=30) or dysplastic (n=30) nevi. For genomic analysis, the BRAF gene mutation (V600E) was evaluated using the real-time polymerase chain reaction.

**Results:**

The prevalence of BRAF gene (V600E) mutation was found as 1 case (3.3%) in congenital and 8 cases (26.7%) in dysplastic nevi indicating the higher prevalence of this mutation in patients with dysplastic nevi (*P*=0.026). Moreover, in the dysplastic nevi group, the presence of BRAF gene mutation (V600E) showed a significant relationship with the severity of dysplasia as the mutation rate was 25% in mild cases, in comparison with 54.5% in moderate dysplasia cases (*P*=0.009).

**Conclusion:**

According to the results, 3.3% of the patients with congenital nevi and 26.7% of the subjects with dysplastic nevi were positive for BRAF V600E mutation. Furthermore, the severity of dysplasia could have a positive relationship with the presence of the mutation.

## Introduction

Numerous epidemiological studies have reported diverse prevalence with a range of 4%-72% for preexisting nevus in cutaneous melanomas (1). A recent review stated that probably 29.1% of melanomas began with a preexisting nevus (2). It has been shown that nevus-associated melanomas are commonly the superficial spreading type and arise in the trunk of younger patients (3-6). However, the relationship between nevus with dysplastic features and melanoma occurrence has not been clearly defined. 

These contradictory results are mostly due to the heterogeneity of the available studies, particularly different histopathological parameters of nevi classification. Moreover, not only patients with large congenital melanocytic nevi have an increased risk of malignant transformation (5%) in their nevi, but also most of the preexisting acquired nevi in melanomas are reported to be intradermal, a type of nevus that usually develops early in life (7, 8). Furthermore, recent evidence found that melanoma and nevus cells in nevus-associated melanomas have similar mutational profiles that might display their common origin or the malignant transformation of a preexisting nevus (9). 

In recent years, V600E mutations in the BRAF gene (v-raf murine sarcoma viral oncogene homolog B) were reported as the most common mutations in melanomas. The BRAF gene encodes a protein called serine/threonine kinase with a basic role in the RAS/mitogen-activated protein kinase (MAPK) pathway (10-14). This signaling cascade is activated by a single-base change in the BRAF gene that converts T to A at nucleotide 1799 substituting a valine for a glutamic acid at codon 600 (V600E). Interestingly, mutations of BRAF have been demonstrated in more than 80% of benign nevi, including the precursors of melanoma (4, 11, 15-17).

According to the literature, the BRAF V600E mutation is frequent not only in acquired benign and dysplastic nevi but also in congenital nevi (18, 19). Moreover, studies indicated that the cases with BRAF V600E expression and congenital features are clinically small nevi and not large, which shows an NRAS mutation instead of BRAF V600E. Small and medium congenital nevi are rarely associated with melanoma transformation. Consequently, these ﬁndings indicate the controversy of the theory that BRAF oncogene activation is an early crucial event in melanoma progression (20).

The BRAF V600E mutation is considered as the most common oncogene and precursor in melanoma and is of remarkable value as a therapeutic target. In addition, the importance of early melanoma detection is clear. Therefore, it is crucial to better define the prevalence of this mutation in dysplastic and congenital nevi. With this background in mind, the present study aimed to identify the incidence and main determinants of a common BRAF mutation (V600K with the transformation of GTG to AAG) in dysplastic and congenital nevi in the Iranian population using the polymerase chain reaction (PCR).

##  Materials and Methods

This cross-sectional retrospective study was conducted on subjects diagnosed with congenital (n=30) and dysplastic (n=30) nevi who referred to Razi Hospital and Imam Khomeini Hospital in Tehran, Iran. The study was approved by the ethical committee of Tehran University of Medical Sciences with the code of IR.TUMS.VCR.REC.1397.107.

According to the histological patterns and clinical evidence, the nevi were categorized into the two groups of congenital or dysplastic. The diagnosis of congenital nevi was confirmed based on the histological features, including (1) the involvement of deep dermal appendages and neurovascular structures, such as hair follicles, sebaceous glands, arrector pili muscles, and blood vessels walls by nevus cells, (2) the extension of nevus cells to the deep dermis and subcutaneous fat, (3) the infiltration of nevus cells between collagen bundles, and (4) a nevus cell–poor subepidermal zone. 

For these nevi with congenital histological features, the medical records were reviewed to confirm their presence at birth. In dysplastic or atypical nevi, the following findings were considered to differentiate from common nevi: 1) an increased number of single melanocytes along the basal layer with the elongation of rete ridges, 2) the cytologic atypia of melanocytes with enlarged hyperchromatic nuclei in the junctional component, 3) a horizontal arrangement of melanocytes, which generally varies in shape from round to spindled and an occasional epithelioid configuration may be identified, 4) a tendency for melanocytes to aggregate into variably sized nests fusing with adjacent rete ridges to produce bridges, 5) the presence of lamellar and concentric dermal fibroplasia, 6) the presence of a lymphocytic infiltrate as patchy or diffuse in the superficial dermis, and 7) the extension of the junctional component of rete ridges beyond the last dermal nest making "shoulders". 

Characteristics of the patients including demographics, medical history, medications, features of nevi, such as the size, number, and site of nevi, as well as exposure to sunlight were collected from the files of patients.

For the genomic analysis of BRAF mutations, paraffin tissue blocks of 5 µm were prepared. One of the sections was examined for nevi cells under Hematoxylin and Eosin (H&E) staining. The other sections were deparaffined and their DNAs were extracted using the salting-out technique. In the next step, the quality of extracted DNAs was investigated by examining the absorbance ratio of 280/260. Next, the PCR Master Mix solution was prepared and finally, the BRAF gene mutation (V600E) was evaluated using the real-time PCR technique. 

In terms of statistical analysis, the results were presented as mean ± standard deviation (SD) for the numerical variables and were summarized as absolute frequencies and percentages for the categorical variables. The normality of data distribution was assessed utilizing the Kolmogorov-Smirnov test. Categorical variables were compared using the chi-square test or Fisher's exact test when more than 20% of the cells had an expected count of less than 5. Moreover, the quantitative variables were compared by the t-test or Mann-Whitney U test. All the data were analyzed using the SPSS software version 16 for windows (SPSS Inc., Chicago, IL). P-value ≤ 0.05 was considered statistically significant.

## Results

Overall, 30 samples with congenital nevi and 30 with dysplastic nevi were included in the current investigation. Among the subjects, 23.3% of the congenital cases and 46.7% of the dysplastic cases were male suggesting that men were more likely to have dysplastic types (*P*=0.047). The mean age of the congenital and dysplastic nevi patients was 26.67±21.88 and 26.87±15.26 years, respectively. The two groups were not significantly different in terms of mean age (*P*=0.956). 

The mean size of the congenital and dysplastic nevi was 1.38±1.04 and 0.48±0.35 cm, respectively. The mean nevi size was significantly higher in the congenital cases (*P*<0.001). In terms of the number of nevus in the congenital group, 86.7% had one nevus, 6.7% had two nevi, and 6.7% had multiple nevi. In the dysplastic nevi group 76.7%, 10%, and 13.3% had one, two, and multiple nevi, respectively. We found that the number of nevi was not significantly different between the two groups (*P*=0.591). 

**Fig. 1:**
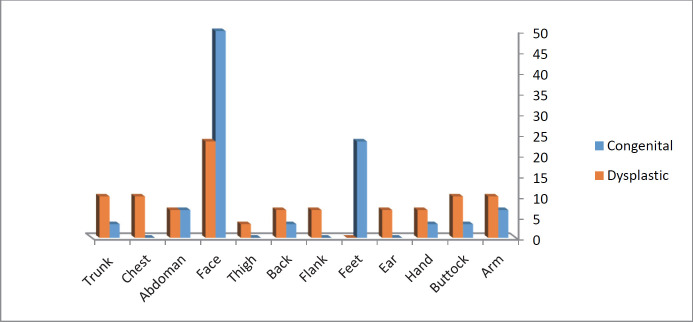
Sites of involvement with the nevi

Regarding the site of involvement (Figure 1), in the group of congenital nevi, the most common sites were face (50%) and legs (23.3%), while in the dysplastic nevi group the most affected sites entailed face (23.3%), arms (10%), hips (10%), trunk (10%), and chest (10%) (*P*=0.036). Moreover, the rate of exposure to sunlight in groups with congenital and dysplastic types was 56.7% and 36.7%, respectively. The results demonstrated that the difference in exposure to sunlight was not significant between the two groups (*P*=0.121).

The prevalence of BRAF V600E mutation was one case (3.3%) in the congenital group and eight cases (26.7%) in the dysplastic group indicating the higher prevalence of this mutation in patients with dysplastic nevi (*P*=0.026). The congenital nevi case positive for BRAF V600E mutation was a 27-years old woman with a congenital nevus sized about 4 cm on the dorsal part of the hand exposed to sunlight. Given that only one patient with the relevant mutation was identified in the congenital nevi group, it was not practically possible to analyze the relevant mutation with other underlying indicators of the patients. 

In the dysplastic nevi group, the comparison of the subgroups with and without BRAF V600E mutation showed no difference in the evaluated parameters, including gender, age, sunlight exposure, and characteristics of the nevi (Table 1). The severity of dysplasia was significantly correlated with the presence of mutation as the rate of mutation was 25.0% in mild dysplasia and 54.5% in moderate dysplasia (*P*=0.009).

**Table 1:** Demographic characteristics of dysplastic nevi group with and without BRAF V600E mutation

Variables	Group with mutation (N=8)	Group without mutation (N=22)	P-value
Female gender	75.0%	45.5%	0.226
Mean age, year	29.50 ± 17.84	25.91 ± 14.55	0.578
Mean size (centimeter)	0.70 ± 0.53	0.53 ± 0.26	0.257
Number of nevus			
One Two Multiple	87.5% 12.5% 0.0%	72.7% 9.0% 18.2%	0.430
Site of involvement Arm Buttock Hand Ear Flank Back Thigh Face Abdomen Chest Trunk	0.0% 12.5% 0.0% 12.5% 12.5% 12.5% 0.0% 50.0% 0.0% 0.0% 13.6%	13.6% 9.1% 9.1% 4.5% 4.5% 4.5% 4.5% 13.6% 9.1% 13.6% 13.6%	0.104
Exposure to sunlight	62.5%	27.3%	0.104

The findings of the current study revealed the prevalence of BRAF V600E mutation as 14.3% in men and 37.5% in women with dysplastic nevi with a significant difference (*P*=0.226). Furthermore, the prevalence of this mutation in cases aged under and over 30 years was 26.3% and 27.3%, respectively. These two age groups did not have a significantly different prevalence (*P*=0.998). 

Furthermore, in patients with nevi smaller than 0.5 cm, the prevalence of BRAF V600E mutation was 16.7% and in cases with nevi larger than 0.5 cm, the prevalence was 33.3%, which were not significantly different (*P*=0.419). Prevalence of the mentioned mutation was 30.4%, 33.3%, and 0% in patients with one, two, and more nevi, respectively. The latter finding showed that the prevalence of this mutation did not have a significant difference between the aforementioned groups (*P*=0.43). 

In patients with sunlight exposure, the prevalence of BRAF V600E mutation was reported as 45.5% and in patients without exposure to sunlight, the prevalence was 15.8% indicating that the two groups were not significantly different (*P*=0.104). The overall prevalence of BRAF V600E mutation in those with buttock, ear, flanks, back, and face involvement was 33.3%, 50%, 50%, 50%, and 57.1%, respectively. According to our results, the difference in prevalence between these groups was not significant (*P*=0.427).

## Discussion

Predicting the transformation of dysplastic or congenital nevi into malignant lesions results in a significant increase in the survival of the patients. This is especially important because melanoma is one of the deadliest skin tumors in humans. Consequently, along with histopathological changes, some laboratory markers and more importantly gene mutations in this field could be very helpful. Among the described gene mutations, mutations in the BRAF gene have recently been taken into consideration and the relationship between BRAF V600E mutation and the transformation of congenital or dysplastic nevus to malignant melanoma has been shown (9-12, 20).

In a recent study, the prevalence of BRAF V600E mutation was evaluated in Iranian patients with pigmented cutaneous neoplasms. They revealed a high intensity of immunostaining for BRAF V600E in 60% of the cases in the malignant melanoma group, while not in other skin neoplasms, such as benign nevus (21). However, the relationship between BRAF V600E mutation and the transformation of a nevus to malignant melanoma remains obscure in the Iranian population. As a result, the current study aimed to evaluate the prevalence of BRAF V600E mutation in patients with dysplastic and congenital nevi. Moreover, the relationship between the occurrence of this mutation and other underlying features of the patients was investigated.

Our results revealed that only 3.3% of the patients with congenital nevi were positive for this mutation indicating the lack of this mutation or its relationship with melanoma development in congenital nevi. Dessars *et al.* reported a low incidence of 15% for BRAF V600E mutation using fluorescence in situ hybridization in cases with congenital nevi (22).

On the other hand, the remarkable prevalence of 26.7% was shown for BRAF V600E mutation in patients with dysplastic nevi. However, the role of this mutation in the transformation of these nevi into melanoma remains questionable. It is noteworthy that in the dysplastic nevi group, the subgroups with and without BRAF V600E mutation had a significant difference in the severity of dysplasia as the rate of mutation was 25% in mild dysplasia and 54.5% in moderate dysplasia cases. However, given the small sample size, it is hard to confirm the role of this mutation in the transformation of dysplastic nevi to melanoma. 

Furthermore, we evaluated the relationship between BRAF V600E mutation and the demographic characteristics of patients, as well as the parameters of nevi, such as size, number, position, and exposure to sunlight. Our results revealed no significant relationship between this mutation and the mentioned variables.

The high prevalence of BRAF V600E mutation in patients with dysplastic nevi was documented in several studies. Tan *et al.* showed the high prevalence of dysplastic nevi in cases with BRAF V600E mutation (23). Saroufim *et al.* described BRAF mutation in 101 out of 125 cases (80.8%) of dysplastic nevi. Contrary to our study, they concluded that the observed mutations were correlated with sunlight exposure (24).

In the study performed by Wu *et al.*, the overall mutation rate was 81% in dysplastic nevi, which is far more than our findings. In line with our study, these authors reported no relationship between the presence of BRAF mutations and exposure to sunlight. However, they found no discrepancy in the mutation rate between the dysplastic and congenital nevi, which is completely inconsistent with the present study (25). 

In another study conducted by Papp *et al.*, 94.4% of the cases with dysplastic nevi and 27.7% with congenital nevi had BRAF mutation, which is higher than our findings (26). A review of the literature reveals that the prevalence of BRAF V600E mutations both in the dysplastic and congenital groups is much lower in our study on the Iranian population than other societies. Consequently, it could be assumed that this mutation marker may not have a causal relationship with the occurrence of melanoma following both types of nevi in our population. However, it is worth mentioning that different investigations have used diverse methods to detect BRAF V600E mutation and there is not a standardized protocol for the detection of this mutation. Therefore, the contradictory results of studies in different ethnicities might be attributed to the application of various detection protocols. As a result, further studies with larger samples of Iranian ethnics using several detection methods are recommended for comparison.

## Conclusion

According to the results of the current study, 3.3% of the patients with congenital nevi and 26.7% of dysplastic nevi cases were positive for BRAF V600E mutation. The severity of dysplasia was positively correlated with the presence of the mutation. However, the mutation showed no significant relationship neither with the demographic characteristics of patients nor with the size, number, site of nevi, and history of exposure to sunlight.
